# Perioperative Blood Loss in Cementless Versus Cemented Total Knee Arthroplasty With Local Tranexamic Acid Administration

**DOI:** 10.7759/cureus.93791

**Published:** 2025-10-03

**Authors:** Daichi Ishimaru, Nobuo Terabayashi, Kazuki Sohmiya, Kazu Matsumoto

**Affiliations:** 1 Orthopaedic Surgery, Gifu Seiryu Hospital, Gifu, JPN; 2 Orthopedics, Gifu Seiryu Hospital, Gifu, JPN

**Keywords:** blood loss, blood transfusion, cemented total knee arthroplasty, cementless total knee arthroplasty, tranexamic acid

## Abstract

Background: Cementless total knee arthroplasty (TKA) has historically been associated with greater perioperative blood loss than cemented TKA. Whether a standardized local-only tranexamic acid (TXA) regimen can mitigate this difference remains unclear.

Objective: To compare perioperative blood loss between cementless and cemented TKA under a standardized local-only TXA protocol in a retrospective cohort, focusing on comparison rather than causation. Secondary outcomes included perioperative hemoglobin (Hb)/hematocrit (Hct) changes (preoperative to postoperative day (POD) 1), intraoperative and perioperative blood loss, transfusion, POD 7 D-dimer, operative time, and complications.

Methods: We retrospectively reviewed 103 consecutive unilateral primary TKAs performed at a single center (cementless, n=55; cemented, n=48) between 2013 and 2020. A standardized local TXA protocol was used in all cases (1 g periarticular before implantation and 1 g intra-articular before closure); no drains were used. Outcomes included operative time, perioperative Hb/Hct change, intraoperative blood loss, total perioperative blood loss estimated by the Gross formula, transfusion, complications, and POD 7 D-dimer. Group comparisons used t-tests with Welch’s correction or Mann-Whitney U tests, and Fisher’s exact tests for categorical variables.

Results: Baseline demographics were comparable between groups. Intraoperative blood loss was similar (cementless 91.4 ± 75.1 mL vs cemented 85.5 ± 111.2 mL; p=0.140), as was perioperative blood loss (154.3 ± 76.9 mL vs 132.9 ± 91.0 mL; p=0.204). No patient required transfusion. POD 7 D-dimer was higher in the cementless group (9.7±4.1 µg/mL vs 7.8±4.4 µg/mL; p=0.002), but no symptomatic venous thromboembolism occurred. Two late surgical-site infections in the cementless group resolved without revision or loosening; no wound complications occurred in either group.

Conclusions: Under a standardized local-only TXA protocol, cementless TKA achieved perioperative blood loss comparable to cemented TKA without increasing symptomatic thromboembolic events. These findings suggest the feasibility of a standardized local TXA protocol for cementless TKA. Importantly, fixation method was fully collinear with implant design (cementless: low contact stress (LCS) limited-box; cemented: posterior-stabilized (PS) open-box), and femoral box geometry can influence bleeding; therefore, results should be interpreted within this implant- and protocol-specific context.

## Introduction

Cemented and cementless total knee arthroplasty (TKA) are established surgical options for managing knee osteoarthritis (OA), offering similar clinical outcomes [1]. Cementless TKA allows for biological fixation and bone preservation but may lead to early component subsidence and has historically been associated with greater perioperative blood loss compared to cemented TKA [1,2]. A recent meta-analysis confirmed significantly higher blood loss in cementless TKA [2].

Tranexamic acid (TXA) administration has emerged as a safe and effective method to reduce perioperative blood loss in TKA, without increasing thromboembolic risk [3]. Although optimal TXA administration protocols remain under debate, local administration has shown favorable outcomes in cemented TKA, including reduced blood loss and improved postoperative recovery [4,5]. However, data on TXA’s efficacy relative to fixation method remain limited.

This study aimed to evaluate whether local TXA administration reduces perioperative blood loss in cementless TKA and to compare outcomes with cemented TKA.

## Materials and methods

Ethics approval and consent to participate

This study was approved by the ethical review board of Gifu Seiryu Hospital (approval number: 071) and conducted in accordance with the principles of the Declaration of Helsinki. All participants were informed of the study details and were given the opportunity to decline participation.

Study design and setting

We conducted a retrospective comparative study at Gifu Seiryu Hospital (Japan), analyzing consecutive unilateral primary TKAs performed between January 2013 and December 2020.

Study objective

The purpose of this retrospective cohort study was to compare perioperative blood loss between cementless and cemented TKA performed under a standardized local-only TXA regimen. Our aim was to assess the comparability of outcomes using effect sizes and 95% confidence intervals (CIs), without implying causation. Secondary objectives were to evaluate perioperative hemoglobin (Hb) and hematocrit (Hct) changes (preoperative to postoperative day (POD) 1), intraoperative and perioperative blood loss estimated using the Gross formula, transfusion requirements, POD 7 D-dimer levels, operative time, and postoperative complications including symptomatic deep vein thrombosis/pulmonary embolism, wound complications, and surgical site infection [6]. An exploratory objective was to consider the potential confounding influence of implant design (low contact stress (LCS) limited/closed box vs. posterior-stabilized (PS) open box) on bleeding outcomes.

Sampling technique

A consecutive sampling approach was used; all eligible cases during the study period were included. In total, 113 primary TKA knees were assessed. After applying the eligibility criteria, exclusions were as follows: In the cementless cohort (initial n=59), bilateral TKA (n=2), missing perioperative data (n=1), and hybrid fixation with tibial cement (n=1); in the cemented cohort (initial n=54), bilateral TKA (n=4), and use of augments/stems/constrained components (n=2). Accordingly, 55 cementless and 48 cemented knees were included in the final analysis (total n=103).

Eligibility criteria

Inclusion criteria were as follows: (1) primary unilateral TKA for OA; (2) PS or cruciate-retaining/mobile-bearing design as per implant system; the cementless cohort received an LCS mobile-bearing with limited box, whereas the cemented cohort received a PS open-box femoral component; (3) periarticular and intra-articular local TXA protocol; (4) available perioperative data (Hb/Hct) on preoperative and POD 1, and D-dimer on POD 7.

**Figure 1:**
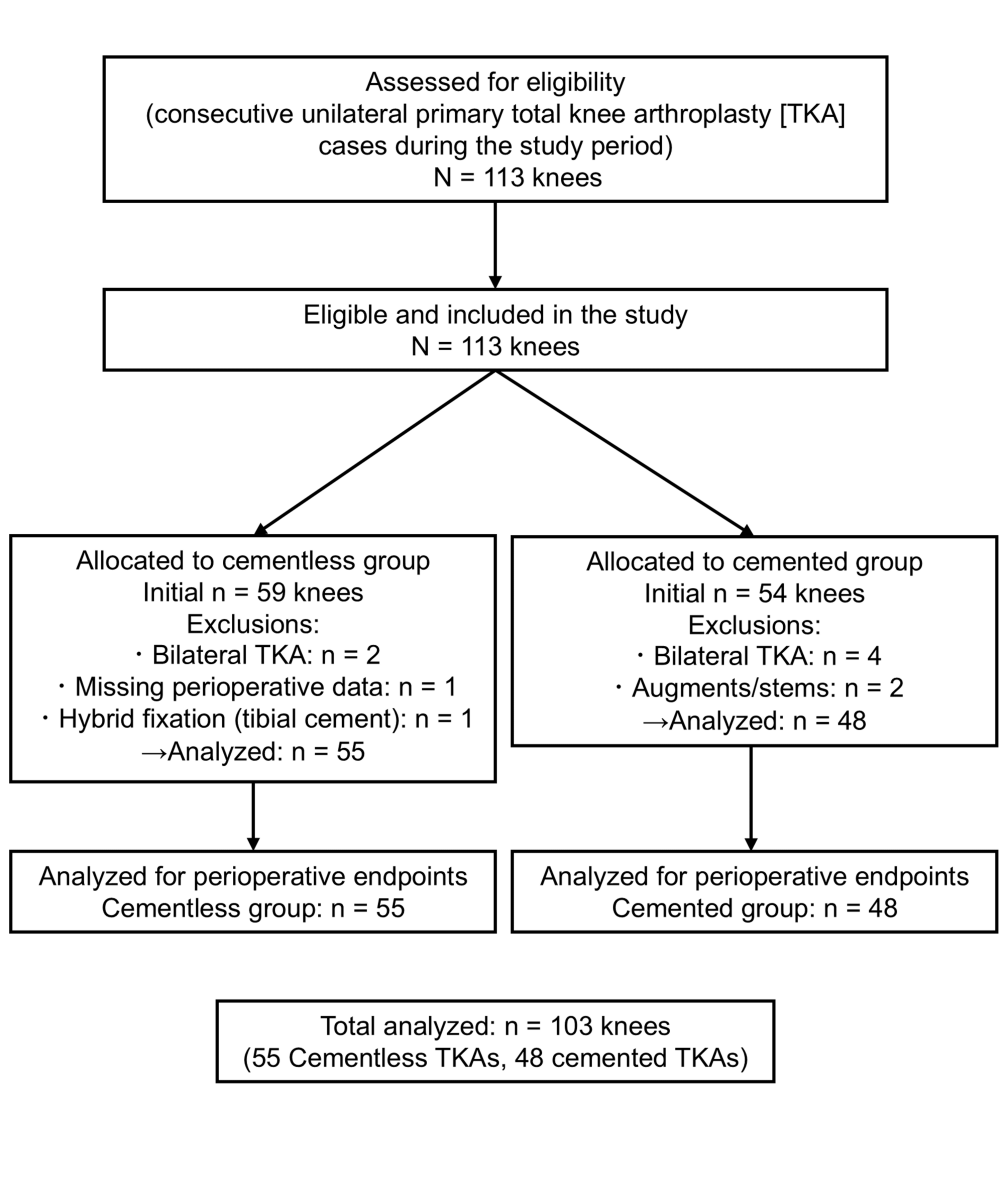
Flow diagram of case inclusion/exclusion and analysis During the study period, 113 primary TKA knees were assessed. After applying the eligibility criteria, 55 cementless knees (LCS limited-box) and 48 cemented knees (Vanguard PS open-box) were included in the final analysis. TKA: Total knee arthroplasty; LCS: Low contact stress; PS: Posterior-stabilized

Exclusion criteria were as follows: (1) simultaneous bilateral TKA; (2) hybrid fixation, augments, stems, or constrained implants; (3) revision TKA; (4) incomplete perioperative data. A flow diagram of case inclusion and exclusion is shown in Figure 1.

Groups and implants

In the cementless cohort, we used the LCS mobile-bearing system (DePuy Synthes, USA), which requires a limited femoral box. In the cemented cohort, we used the Vanguard PS design (Zimmer Biomet, USA) with an open-box femoral component. In this dataset, fixation method and implant design were completely collinear (cementless: LCS limited box; cemented: PS open box). Because femoral box geometry can influence bleeding, this represents a major confounder, which we highlight as a primary limitation in the Discussion section.

Surgical procedure and local TXA administration protocol

**Figure 2:**
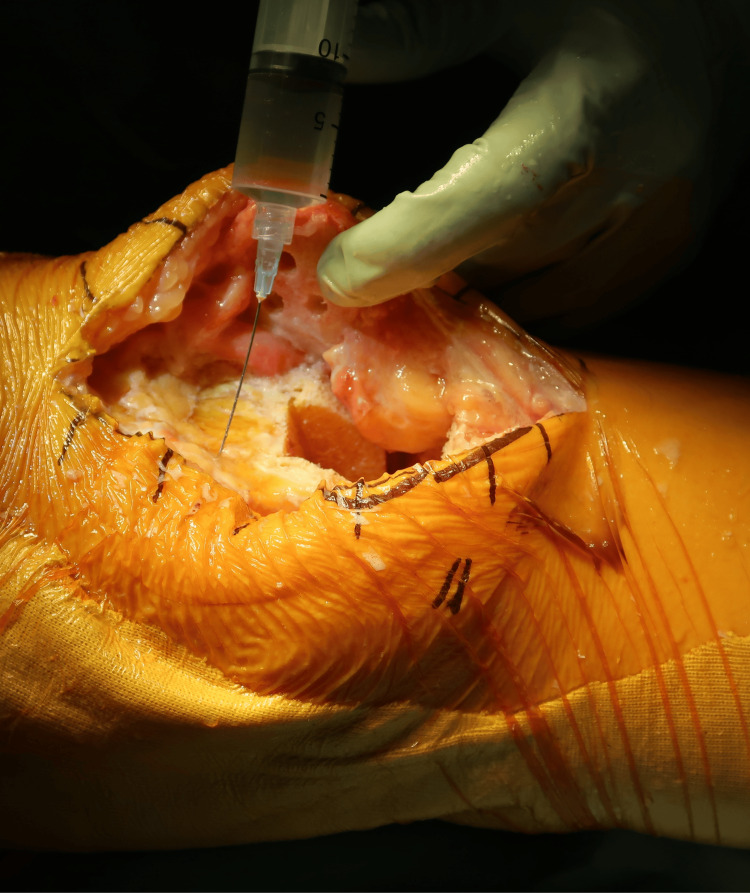
Periarticular Injection of TXA around the joint capsule Periarticular injection of TXA was performed into the anterior capsule, medial and lateral gutters, and retinacula before capsular closure; the remaining dose was administered intra-articularly after closure. TXA: Tranexamic acid

All surgeries were performed via a medial parapatellar approach. Bone cuts were made using extramedullary alignment guides and a gap-balancing technique under tourniquet control. After tourniquet release, meticulous hemostasis was achieved. In accordance with a previously described protocol, 1 g of TXA was infiltrated periarticularly before implantation, irrespective of fixation method, targeting the posterior capsule (posteromedial and posterolateral regions), the infrapatellar fat pad and adjacent synovium, and the deep layer beneath the arthrotomy along the medial parapatellar approach, including cut edges of the capsule/retinaculum and the vastus medialis (Figure 2) [4]. An additional 1 g of TXA was injected intra-articularly before closure. Each 1-g dose of TXA was diluted to a total volume of 10 mL in normal saline. No surgical drains were used.

Perioperative care protocol

All cases followed a standardized perioperative pathway. General or spinal anesthesia was administered at the anesthesiologist’s discretion. Prophylactic antibiotics were given according to institutional policy (administered within 30 minutes before incision, with postoperative doses per protocol). A tourniquet was used during bony preparation and released prior to hemostasis. No surgical drains were used. Thromboprophylaxis consisted of early mobilization and mechanical prophylaxis (graduated compression and intermittent pneumatic compression); pharmacologic prophylaxis was used according to the institutional risk-stratification protocol. Patients were mobilized under physiotherapy on POD1

Clinical outcomes and measurement methods

We analyzed operative time, preoperative and POD 1 Hb/Hct levels, intraoperative blood loss, perioperative blood loss, transfusion requirements, POD 7 D-dimer levels, and complications including symptomatic venous thromboembolic events. 

Intraoperative blood loss was defined as the suction canister volume minus irrigation fluid plus the net weight gain of surgical gauze (wet minus dry). Perioperative blood loss was estimated using the Gross formula [6].

Statistical analysis

The Shapiro-Wilk test was applied to assess the normality of data distribution for each continuous variable within groups. Variables that satisfied the assumption of normality (p > 0.05) were analyzed using parametric tests, specifically unpaired t-tests with Welch’s correction for unequal variances. Variables that did not meet the assumption of normality (p ≤ 0.05) were analyzed using non-parametric tests, specifically the Mann-Whitney U test. Categorical variables, including sex and primary diagnosis, were compared between groups using Fisher’s exact test. For all statistical tests, two-tailed p-values < 0.05 were considered statistically significant. The corresponding test statistics (t-value, U-value, or Fisher’s exact p-value) were reported alongside the p-value. Statistical analyses were performed using GraphPad Prism version 5.02 (GraphPad Software, Inc., USA). In addition, Python (version 3.11; SciPy library) was used to calculate test statistics (e.g., U values) that were not directly provided by GraphPad Prism, to ensure compliance with journal requirements.

## Results

Demographic characteristics were comparable between groups (Table 1).

**Table 1:** Demographic characteristics of patients undergoing cementless versus cemented TKA Values are presented as mean ± SD. Mean differences and 95% CIs were calculated using Welch’s method unless otherwise indicated TKA: Total knee arthroplasty; CI: Confidence interval; OA: Osteoarthritis; RA: Rheumatoid arthritis

	Cementless Group (N=55)	Cemented Group (N=48)	Test Used	Test Statistic	p-Value	Mean Difference (95% CI)
Sex (Male/Female)	13/42	11/37	Fisher’s exact test	N/A	1.00	N/A
Diagnosis (OA/RA)	52/3	43/5	Fisher’s exact test	N/A	0.468	N/A
Age (y)	75.1±6.4	74.2±7.9	Mann–Whitney U	U=1353.5	0.827	0.9 (-2.3 to 4.1)
Height (cm)	154.2±7.8	152.7±7.4	Mann–Whitney U	U=1513.5	0.201	1.5 (-0.8 to 3.8)
Weight (kg)	59.7±10.3	59.8±10.1	Welch t-test	t=0.056	0.935	-0.1 (-3.3 to 3.1)
BMI	25.0±3.5	25.6±3.5	Welch t-test	t=0.797	0.427	-0.6 (-2.0 to 0.9)

Sex distribution (male/female: 13/42 vs. 11/37; p=1.000, Fisher’s exact test) and primary diagnosis (OA/RA: 52/3 vs. 43/5; p=0.468, Fisher’s exact test) did not differ between groups. Age was 75.1 ± 6.4 years in the cementless group and 74.2 ± 7.9 years in the cemented group (U=1353.5, p=0.827; mean difference=0.9 years, 95% CI=-2.3 to 4.1). Height was 154.2 ± 7.8 cm in the cementless group and 152.7 ± 7.4 cm in the cemented group (t=1.28, p=0.201; mean difference=1.5 cm, 95% CI=-0.8 to 3.8). Weight was 59.7 ± 10.3 kg in the cementless group and 59.8 ± 10.1 kg in the cemented group (t=0.056, p=0.935; mean difference=-0.1 kg, 95% CI=-3.3 to 3.1). BMI was 25.0 ± 3.5 in the cementless group and 25.6 ± 3.5 in the cemented group (t=-0.797, p=0.427; mean difference=-0.6, 95% CI=-2.0 to 0.9). Hb and Hct values decreased similarly in both groups (Table 2).

**Table 2:** Preoperative and postoperative Hb and Hct values Values are presented as mean ± SD. Mean differences and 95% CI were calculated using Welch’s method. CI: Confidence interval; Hb: Hemoglobin; Hct: Hematocrit; POD: Postoperative day

	Cementless Group (N=55)	Cemented Group (N=48)	Test Used	Test Statistic	p-Value	Mean Difference (95% CI)
Hb (g/dL)						
Preoperative	12.6±1.3	12.3±1.3	Welch t-test	t=1.634	0.105	0.3 (-0.1 to 0.7)
POD1	11.4±1.2	11.1±1.1	Welch t-test	t=1.141	0.256	0.3 (-0.2 to 0.8)
Hct (%)						
Preoperative	37.7±3.3	36.4±3.6	Welch t-test	t=1.918	0.058	1.3 (-0.05 to 2.7)
POD 1	33.4±3.0	32.6±2.9	Welch t-test	t=1.436	0.154	0.8 (-0.3 to 1.9)

Preoperative Hb was 12.8 ± 1.3 g/dL in the cementless group and 12.3 ± 1.3 g/dL in the cemented group (t=1.634, p=0.105; mean difference=0.3 g/dL, 95% CI=-0.1 to 0.7). On POD 1, Hb was 11.4 ± 1.2 g/dL in the cementless group and 11.1 ± 1.1 g/dL in the cemented group (t=1.141, p=0.256; mean difference=0.3 g/dL, 95% CI=-0.2 to 0.8). Preoperative Hct was 37.7 ± 3.3% in the cementless group and 36.4 ± 3.6% in the cemented group (t=1.918, p=0.058; mean difference=1.3%, 95% CI=-0.05 to 2.7), and POD 1 Hct was 33.4 ± 3.0% in the cementless group and 32.6 ± 2.9% in the cemented group (t=1.436, p=0.154; mean difference=0.8%, 95% CI=-0.3 to 1.9). None of these differences reached statistical significance.

Blood loss parameters are summarized in Table 3.

**Table 3:** Intraoperative blood loss, perioperative blood loss, and POD 7 D-dimer values Values are presented as mean ± SD. Mean differences and 95% CI were calculated using Welch’s method. POD: Postoperative day; CI: Confidence interval

	Cementless Group (N=55)	Cemented Group (N=48)	Test Used	Test Statistic	p-Value	Mean Difference (95% CI)
Intraoperative blood loss (mL)	91.4 ± 75.1	85.4 ± 111.0	Mann–Whitney U	U=1543.5	0.140	5.9 (-31.9 to 43.7)
Perioperative blood loss (mL)	154.3 ± 76.9	132.9 ± 91.0	Welch t-test	t=1.279	0.204	21.4 (-11.8 to 54.6)
POD 7 D-dimer (µg/mL)	9.7 ± 4.1	7.8 ± 4.4	Mann–Whitney U	U=1438.5	0.002	N/A

Intraoperative blood loss was 91.4 ± 75.1 mL in the cementless group and 85.5 ± 111.2 mL in the cemented group (U=1543.5, p=0.140; mean difference=5.9 mL, 95% CI=-31.9 to 43.7). Perioperative blood loss, estimated using the Gross formula, was 154.3 ± 76.9 in the cementless group and 132.9 ± 91.0 mL in the cemented group (t=1.279, p=0.204; mean difference=21.4 mL, 95% CI=-11.8 to 54.6). No patients required transfusion. D-dimer levels on POD 7 were significantly higher in the cementless group (9.7 ± 4.1 µg/mL vs. 7.8 ± 4.4 µg/mL; U=1438.5, p=0.002), although no symptomatic deep vein thrombosis was observed. Two patients in the cementless group developed late surgical site infections, both of which were successfully managed without revision or loosening. No wound complications were noted in either group.

## Discussion

Our results indicate that with local TXA administration, perioperative blood loss in cementless TKA is comparable to that in cemented TKA. No thromboembolic complications were observed, suggesting the safety of this approach. This observation is consistent with recent large-scale analyses indicating that perioperative TXA does not increase thromboembolic complications in arthroplasty, including in high-risk or prior-VTE populations [3,7,8]. The safety of TXA has been consistently demonstrated regardless of the route of administration-intravenous, topical, or combined-without increasing thromboembolic complications [3,7,9-11]. In addition, topical use has been linked to improved recovery outcomes [4,5]. This is in line with our observation that local TXA use did not increase adverse events in either group. In contrast to other topical hemostatic agents, a recent cohort study reported that intra-articular microporous polysaccharide hemospheres (MPH) powder was less effective than TXA in reducing early postoperative drainage and maintaining Hb levels, with notably higher transfusion rates in simultaneous bilateral TKA [12]. Although that cohort used drains and cemented PS implants with intra-articular TXA delivered via the drain, the direction of effect supports the superiority of TXA among topical strategies.

Although cemented fixation offers mechanical stability and may reduce early subsidence, cementless fixation avoids cement-related complications and promotes bone ingrowth [13,14]. These observations suggest that, under standardized blood-management protocols, any historical disadvantage of cementless fixation regarding blood loss may be reduced. This interpretation should be considered in light of the retrospective design and the potential confounding effect of implant geometry. In addition, a Level-I randomized trial using the same implant design found equivalent midterm survivorship and patient-reported outcomes between cementless and cemented TKA, reinforcing that fixation method per se is unlikely to drive perioperative differences under standardized care [15].

Historically, cementless TKA has been associated with greater blood loss [16]. A systematic review reported 140-655 mL higher blood loss in cementless cases, attributed to the absence of a cement barrier [2]. However, our findings suggest local TXA administration mitigates this difference. Cao et al. similarly demonstrated comparable blood loss between fixation types with intravenous TXA [17]. Recent propensity score-matched studies have demonstrated that cementless TKA does not result in greater perioperative blood loss or transfusion requirements compared with cemented TKA when performed under a contemporary patient blood management protocol including both systemic and topical TXA administration [18,19]. Unlike these previous studies that applied combined intravenous and topical TXA, our study demonstrated that local TXA administration alone (periarticular and intra-articular injections) resulted in comparable perioperative blood loss (see effect sizes and 95% CIs in Table 3). This study was underpowered to conclude equivalence or non-inferiority. Our interpretation is supported by recent bilateral within-patient data demonstrating that peri-articular TXA (2 g), either alone or combined with intra-articular injection, reduced postoperative drainage compared with intra-articular injection alone, without increasing complications [20]. Collectively, this finding highlights the effectiveness of a local-only TXA protocol and suggests that systemic administration may not be mandatory to achieve adequate blood conservation in cementless TKA.

Implant design differences may also have contributed to our findings. Notably, femoral box geometry can itself affect bleeding: closed-box prostheses have been shown to reduce total blood loss versus open-box designs by approximately 173 mL on pooled analysis, without differences in transfusion or length of stay [21]. In our cohort, the cementless group used the LCS mobile-bearing, which requires relatively limited box resection, whereas the cemented group used the Vanguard PS design with an open-box femoral component. This design discrepancy represents a major confounder, as it may account for much of the observed difference in blood loss independent of fixation method. In PS prostheses with open-box femoral components, the open-box design has been associated with greater blood loss [22]. Taken together, opposing design- and fixation-related influences may have partially offset each other between groups: In the cementless LCS cohort, the absence of cement (potentially increasing bleeding) may have been counterbalanced by limited/closed femoral box resection (reducing bleeding), whereas in the cemented Vanguard PS cohort, cement sealing of bone surfaces (reducing bleeding) may have been counterbalanced by the open-box geometry (increasing bleeding). Topical TXA may have further attenuated bleeding in both groups, contributing to the small between-group differences. These considerations are exploratory and should be interpreted with caution given the retrospective design, modest sample size, and the possibility of residual confounding.

Limitations

This study has several limitations. First, implant design was fully collinear with fixation method (cementless: LCS limited-box mobile-bearing; cemented: PS open-box femur). Because femoral box geometry can influence bleeding, this constitutes a major confounder, and effects cannot be attributed to fixation per se. Findings should, therefore, be interpreted within the implant- and pathway-specific context used here. Second, this was a single-center retrospective cohort with a modest sample size and no apriori sample size/power calculation; as with any retrospective design, the cohort is susceptible to selection bias and unmeasured confounding. The study was not powered to establish equivalence or non-inferiority, so we report effect sizes with 95% CIs to aid interpretation. Third, measurement variability is possible: Intraoperative blood loss was derived from suction canister volume minus irrigation fluid plus gauze net weight, and total perioperative blood loss from the Gross formula based on Hb/Hct at specified time points-methods with inherent error. Fourth, procedures were performed by six surgeons, which may introduce variability, although all were experienced in TKA and followed a standardized protocol. Fifth, functional outcomes (e.g., pain, swelling, range of motion) were not assessed. Sixth, routine duplex ultrasonography was not performed; asymptomatic venous thromboembolic events may therefore have been missed, and POD 7 D-dimer can be influenced by postoperative inflammation and bone metabolism. Seventh, follow-up was short-term and focused on perioperative outcomes (e.g., POD 1 hemoglobin/hematocrit and POD 7 D-dimer), precluding assessment of longer-term complications, implant survivorship, or functional recovery. Prospective studies with apriori power calculations and longer follow-up are warranted.

## Conclusions

Our findings suggest that, under a standardized local TXA protocol, cementless TKA may achieve perioperative blood loss similar to cemented fixation. This strategy may mitigate a traditional drawback of cementless fixation without increasing symptomatic thromboembolic events. These findings suggest the feasibility of clinical use of cementless TKA when coupled with standardized local TXA protocols. Importantly, a local-only TXA regimen is simple and readily implementable across practice settings, which may be useful for patients with relative contraindications to systemic TXA. While this single-center retrospective study has inherent limitations, the internal consistency across outcomes suggests practical robustness within the described perioperative pathway. Future prospective studies should test clinically meaningful noninferiority margins for blood loss and examine how implant geometry interacts with topical TXA to optimize hemostasis.
